# Supplementary material for: Genome analysis of the esca-associated Basidiomycetes Fomitiporia mediterranea, Fomitiporia polymorpha, Inonotus vitis, and Tropicoporus texanus reveals virulence factor repertoires characteristic of white-rot fungi
Source: G3 (Bethesda). 2024 Aug 14;14(10):jkae189. doi: 10.1093/g3journal/jkae189 (PMC11457069; doi:10.1093/g3journal/jkae189)
Supplement: jkae189_Supplementary_Data [file jkae189_supplementary_data.zip › File_S1_G3-2024-405182.pdf]

Genome analysis of the esca-associated Basidiomycetes *Fomitiporia mediterranea*, *Fomitiporia polymorpha*, *Inonotus vitis*, and *Tropicoporus texanus* reveals virulence factor repertoires characteristic of white-rot fungi

Jadran F. Garcia[1], Rosa Figueroa-Balderas[1], Gwenaëlle Comont[2], Chloé E. L. Delmas[2], Kendra Baumgartner[3] and Dario Cantu[1,4*].

Supplemental file1

Table S1. Number of contigs with telomeric repeats in the genomes assemblies.

Species	Isolate	Assembly	Number of contigs	Telomere at both ends	Telomere only at the start	Telomere only at the end
Fomitiporia mediterranea	PHCO36	Hap 1	58	7	3	6
Fomitiporia polymorpha	WFB1	Primary	32	2	2	6
Inonotus vitis	OC1	Primary	54	1	10	13
Tropicoporus texanus	TX9	Primary	41	0	9	6

Table S2. Isolate and reference of the species used for the phylogeny and comparative analysis

Species	Isolate	Reference	Accession link
Saccharomyces cerevisiae	S288C-R64-4-1_20230830	https://www.yeastgenome.org	http://sgd-archive.yeastgenome.org/sequence/S288C_reference/genome_releases/
Botrytis cinerea	v1.0	(Amselem et al. 2011)	https://mycocosm.jgi.doe.gov/Botci1/Botci1.home.html
Fusarium oxysporum f. sp. *lycopersici*	4287 v2	(Ma et al. 2010)	https://mycocosm.jgi.doe.gov/Fusox2/Fusox2.home.html
Phaeomoniella chlamydospora	UCRPC4	(Morales-Cruz et al. 2015)	https://mycocosm.jgi.doe.gov/Phach1/Phach1.home.html
Botryosphaeria dothidea	0053	(Garcia et al. 2021)	https://zenodo.org/records/4417445
Neofusicoccum parvum	UCD646So	(Massonnet et al. 2018)	https://zenodo.org/records/8310403
Fomitiporia mediterranea	PHCO36	(Laveau et al. 2009), this study	https://zenodo.org/records/10957629
Fomitiporia polymorpha	WFB1	(Brown et al. 2020), this study	https://zenodo.org/records/10957629
Inonotus vitis	OC1	(Brown et al. 2020), this study	https://zenodo.org/records/10957629
Tropicoporus texanus	TX9	(Brown et al. 2020), this study	https://zenodo.org/records/10957629
Stereum hirsutum	FP-91666 SS1 v1.0	(Floudas et al. 2012)	https://mycocosm.jgi.doe.gov/Stehi1/Stehi1.home.html
Gloeophyllum trabeum	v1.0	(Floudas et al. 2012)	https://mycocosm.jgi.doe.gov/Glotr1_1/Glotr1_1.home.html
Pleurotus ostreatus	PC15 v2.0	(Riley et al. 2014)	https://mycocosm.jgi.doe.gov/PleosPC15_2/PleosPC15_2.home.html
Serpula lacrymans	S7.9 v2.0	(Eastwood et al. 2011)	https://mycocosm.jgi.doe.gov/SerlaS7_9_2/SerlaS7_9_2.home.html
Trametes versicolor	v1.0	(Floudas et al. 2012)	https://mycocosm.jgi.doe.gov/Trave1/Trave1.home.html
Postia placenta	MAD-698-R-SB12 v1.0	(Gaskell et al. 2017)	https://mycocosm.jgi.doe.gov/PosplRSB12_1/PosplRSB12_1.home.html
Daedalea quercina	v1.0	(Nagy et al. 2016)	https://mycocosm.jgi.doe.gov/Daequ1/Daequ1.home.html
Fomitopsis schrenkii	FP-58527 SS1 v3.0	(Floudas et al. 2012)	https://mycocosm.jgi.doe.gov/Fompi3/Fompi3.home.html

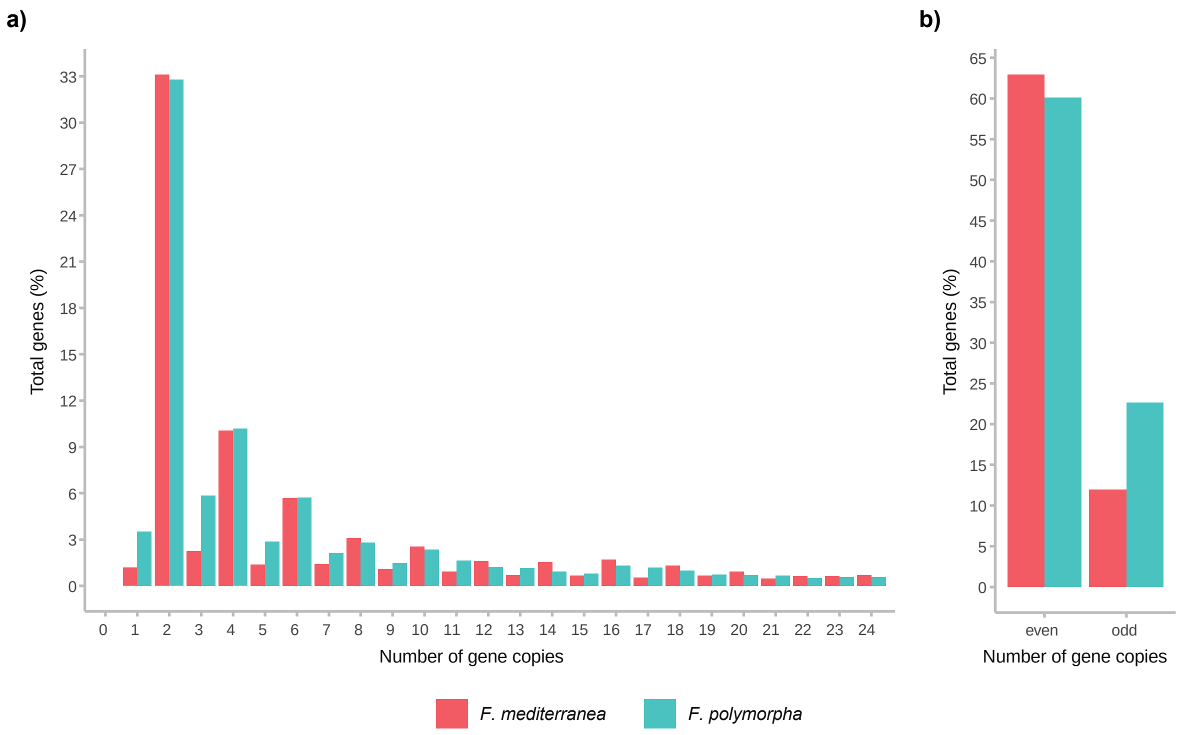

Figure S1. Gene copy number analysis of *F. mediterranea* and *F. polymorpha*

The figure shows a comparison of the gene copy number in the diploid genome of *F. mediterranea* and the full assembly of *F. polymorpha*. (**a**), proportion of total genes per species with different copy numbers. (**b**), proportion of total genes per species with even or odd copy numbers.

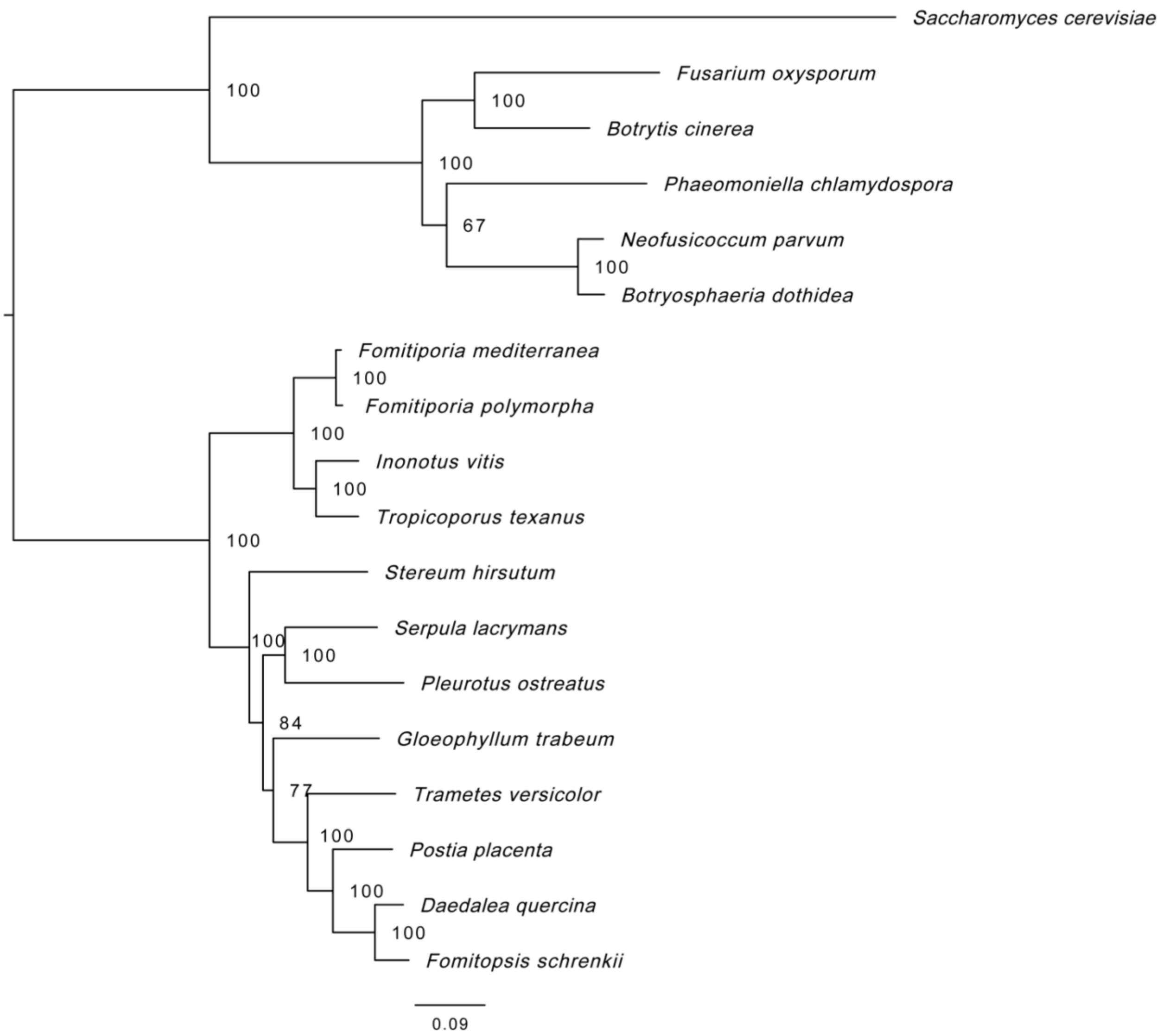

Figure S2. Maximum likelihood phylogenetic tree of the species in the study.

The tree was constructed with a set of single-copy orthologs of the species in the study.

References

Amselem, J., Cuomo, C. A., Kan, J. A. L. van, Viaud, M., Benito, E. P., Couloux, A., et al. 2011. Genomic Analysis of the Necrotrophic Fungal Pathogens Sclerotinia sclerotiorum and Botrytis cinerea. PLOS Genetics. 7:e1002230.

Brown, A. A., Lawrence, D. P., and Baumgartner, K. 2020. Role of basidiomycete fungi in the grapevine trunk disease esca. Plant Pathology. 69:205–220.

Eastwood, D. C., Floudas, D., Binder, M., Majcherczyk, A., Schneider, P., Aerts, A., et al. 2011. The Plant Cell Wall–Decomposing Machinery Underlies the Functional Diversity of Forest Fungi. Science. 333:762–765.

Floudas, D., Binder, M., Riley, R., Barry, K., Blanchette, R. A., Henrissat, B., et al. 2012. The Paleozoic Origin of Enzymatic Lignin Decomposition Reconstructed from 31 Fungal Genomes. Science. 336:1715–1719.

Garcia, J. F., Lawrence, D. P., Morales-Cruz, A., Travadon, R., Minio, A., Hernandez-Martinez, R., et al. 2021. Phylogenomics of Plant-Associated Botryosphaeriaceae Species. Frontiers in Microbiology. 12:587.

Gaskell, J., Kersten, P., Larrondo, L. F., Canessa, P., Martinez, D., Hibbett, D., et al. 2017. Draft genome sequence of a monokaryotic model brown-rot fungus *Postia (Rhodonia) placenta* SB12. Genomics Data. 14:21–23.

Laveau, C., Letouze, A., Louvet, G., Bastien, S., and Guérin-Dubrana, L. 2009. Differential aggressiveness of fungi implicated in esca and associated diseases of grapevine in France. Phytopathologia Mediterranea. 48:32–46.

Ma, L.-J., van der Does, H. C., Borkovich, K. A., Coleman, J. J., Daboussi, M.-J., Di Pietro, A., et al. 2010. Comparative genomics reveals mobile pathogenicity chromosomes in Fusarium. Nature. 464:367–373.

Massonnet, M., Morales-Cruz, A., Figueroa-Balderas, R., Lawrence, D. P., Baumgartner, K., and Cantu, D. 2018. Condition-dependent co-regulation of genomic clusters of virulence factors in the grapevine trunk pathogen Neofusicoccum parvum. Molecular Plant Pathology. 19:21–34.

Morales-Cruz, A., Amrine, K. C., Blanco-Ulate, B., Lawrence, D. P., Travadon, R., Rolshausen, P. E., et al. 2015. Distinctive expansion of gene families associated with plant cell wall degradation, secondary metabolism, and nutrient uptake in the genomes of grapevine trunk pathogens. BMC Genomics. 16:1–22.

Nagy, L. G., Riley, R., Tritt, A., Adam, C., Daum, C., Floudas, D., et al. 2016. Comparative Genomics of Early-Diverging Mushroom-Forming Fungi Provides Insights into the Origins of Lignocellulose Decay Capabilities. Molecular Biology and Evolution. 33:959–970.

Riley, R., Salamov, A. A., Brown, D. W., Nagy, L. G., Floudas, D., Held, B. W., et al. 2014. Extensive sampling of basidiomycete genomes demonstrates inadequacy of the white-rot/brown-

rot paradigm for wood decay fungi. Proceedings of the National Academy of Sciences. 111:9923–9928.
