# Supplementary material for: Genome analysis of the esca-associated Basidiomycetes Fomitiporia mediterranea, Fomitiporia polymorpha, Inonotus vitis, and Tropicoporus texanus reveals virulence factor repertoires characteristic of white-rot fungi
Source: G3 (Bethesda). 2024 Aug 14;14(10):jkae189. doi: 10.1093/g3journal/jkae189 (PMC11457069; doi:10.1093/g3journal/jkae189)
Supplement: jkae189_Supplementary_Data [file jkae189_supplementary_data.zip › File_S2_G3-2024-405182.html]

Genome-analysis-of-the-esca-associated-Basidiomycetes-Fomitiporia-mediterranea,-Fomitiporia-polymorpha,-Inonotus-vitis,-and-Tropicoporus-texanus-reveals-virulence-factor-repertoires-characteristic-of-white-rot-fungi.


# **Genome analysis of the esca-associated Basidiomycetes *Fomitiporia mediterranea*, *Fomitiporia polymorpha*, *Inonotus vitis*, and *Tropicoporus texanus* reveals virulence factor repertoires characteristic of white-rot fungi.**

# Genome assembly

### DATA subsample

```
# CLR subreads were subsampled to retain 25% of the total reads (The reads belonged to species *Fomitiporia polymorpha*, *Inonotus vitis*, and *Tropicoporus texanus*)
for i in $(cat list.CLR.species.txt); do smrtlink_8.0.0.79519/install/smrtlink-release_8.0.0.79519/bundles/smrttools/install/smrttools-release_8.0.0.79510/private/pacbio/pythonpkgs/pbcoretools/bin wrap/bamsieve --percentage 25 --seed 1234 ${i}subreads. bam ${i}_subset_25_subreads.bam; done &

# Subsampled reads were also converted to fasta
for i in $(cat list.CLR.species.txt); do bam2fasta -c 4 -o ${i}_subset_25_subreads -j 20 ${i}_subset_25_subreads.bam ; done &

# Hifi Data were subsampled to keep reads between 15kbp to 25kbp
awk 'BEGIN {FS = "\n"; RS = ">"} NR > 1 {header = $1; seq = ""; for (i = 2; i <= NF; i++) seq = seq $i; if (length(seq) >= 15000 && length(seq) <= 25000) print ">" header "\n" seq}' Fmediterranea.hifi.fasta > Fmediterranea.hifi.15000-25000.fasta.gz &
```

### Genome assemblies

```
#Assembly of CLR data with Canu v.2.2

canu -d TX9_25 -p TX9_25 corOutCoverage=200 "batOptions=-dg 3 -db 3 -dr 1 -ca 500 -cp 50" maxMemory=64 genomeSize=60m useGrid=false -pacbio /group/darcantugrp2/Projects/Basidios_genomes/00_data/25_percentage/TX9_CLR_subset_25.subreads.fasta.gz &

canu -d OC1_25 -p OC1_25 corOutCoverage=200 "batOptions=-dg 3 -db 3 -dr 1 -ca 500 -cp 50" maxMemory=64 genomeSize=60m useGrid=false -pacbio /group/darcantugrp2/Projects/Basidios_genomes/00_data/25_percentage/OC1_CLR_subset_25.subreads.fasta.gz &

canu -d WFB1_25 -p WFB1_25 corOutCoverage=200 "batOptions=-dg 3 -db 3 -dr 1 -ca 500 -cp 50" maxMemory=64 genomeSize=60m useGrid=false -pacbio /group/darcantugrp2/Projects/Basidios_genomes/00_data/25_percentage/WFB1_CLR_subset_25.subreads.fasta.gz &


#Assembly of HiFi data with Hifiasm v.0.19.5-r587. This genome did not need polishing, and it was divided into two haplotypes by the software.
hifiasm-0.19.5-r587/hifiasm/hifiasm -o Fmed.hifiasm.a3_k41_w51_f0_r5_s0.8_D10_N10_n7_m10000000 -t 24 -a 3 -k 41 -w 51 -f 0 -r 5 -s 0.8 -D 10 -N 10 -n 7 -m 10000000 Fmediterranea.hifi.15000-25000.fasta.gz > Fmed.hifiasm.a3_k41_w51_f0_r5_s0.8_D10_N10_n7_m10000000.log &
```

### Polishing of CLR  genomes (Canu assembled)

```
# Subreads were mapped to the raw CLR assemblies using pbmm2 v.0.8.1

for i in $(cat list.CLR.species.txt); do pbmm2 index ${i}.contigs.fasta polish/${i}_25.contigs.mmi --preset SUBREAD ; done &

for i in $(cat list.CLR.species.txt); do pbmm2 align polish/${i}_25.contigs.mmi 00_data/${i}_CLR_subset_25.subreads.bam polish/${i}_25.contigs_movie.bam --preset SUBREAD --sort -j 32 -J 8 --sort-memory 40G --log-level INFO ; done

# Raw CLR assemblies were polished with arrow algorithm within gccp v2.02
for i in $(cat list.CLR.species.txt); do gcpp -r ${i}_25.contigs.fasta -o polish/${i}_25.contigs.P1.fasta,polish/${i}_25.contigs.P1.gff,polish/${i}_25.contigs.P1.vcf --algorithm=arrow -j 48 polish/${i}_25.contigs_movie.bam ; done &
```

### *I. vitis* and *T. texanus* genome splitting (primary and haplotigs)

```
# Duplication was checked using BUSCO v.5.6.1 with the basidiomycota_odb10 database
cd polish/
for i in $(cat list.CLR.species.txt); do busco -i ${i}_25.contigs.P1.fasta -l fungi_odb10 -o ${i}_25_P1_BUSCO -m genome ; done & 

## Step 1: After confirming high duplication levels, genomes of Inonotus vitis and T. texanus were split using the pipeline purge_haplotigs following the instruction at [https://bitbucket.org/mroachawri/purge_haplotigs/src/master/].
cd ..
for i in OC1 TX9 ; do minimap2 -t 40 -a -xmap-pb polish/${i}_25.contigs.P1.fasta ${i}_25.correctedReads.fasta.gz --secondary=no | samtools sort -m 2G -o purge_haplotigs/${i}_25_aligned.bam; done &

for i in OC1 TX9 ; do purge_haplotigs  hist  -b purge_haplotigs/${i}_25_aligned.bam  -g polish/${i}_25.contigs.P1.fasta -t 40 -d 500 2>purge_haplotigs/${i}_hist.log ; done &

## Step 2: After selecting the appropriate cutoffs for each species based on the histogram.
purge_haplotigs  cov  -i purge_haplotigs/TX9_25_aligned.bam.gencov  -l 25  -m 150  -h 230 -o purge_haplotigs/TX9_25_coverage_stats.csv -j 99  -s 80 2>cov.log &

purge_haplotigs  cov  -i purge_haplotigs/OC1_25_aligned.bam.gencov  -l 25  -m 135  -h 230 -o purge_haplotigs/OC1_25_coverage_stats.csv -j 80  -s 80 2>cov.log &

## Step 3: split genome into primary and haplotigs.
for i in OC1 TX9; do purge_haplotigs purge  -g polish/${i}_25.contigs.P1.fasta  -c purge_haplotigs/${i}_25_coverage_stats.csv -t 20 -o ${i}_25_PH -d -b purge_haplotigs/${i}_25_aligned.bam 2>purge_haplotigs/${i}_25_purge.log; done &
```

### *F. polymorpha* genome assembly checks

```
# After confirming high duplication levels and inconsistent results with the purge_haplotigs pipeline, external contamination was checked using ContScout.  
### Prepare directories.
mkdir ContScout
mkdir ContScout/protein_seq ContScout/annotation_data

### Since the gene annotation was required as input, the gene annotation pipeline presented later in this report was used to obtain such files and place them in the  "ContScout/protein_seq" and "ContScout/annotation_data."
cp WFB1_full_canu_v0.1_br_v1_PROT.fasta ContScout/protein_seq/
cp WFB1_full_canu_v0.1_br_v1.gtf ContScout/annotation_data/

### Format GFF3 to meet requirements.
awk '{ if ($3 == "mRNA") print $1 "\t" $2 "\tgene\t" $4 "\t" $5 "\t" $6 "\t.\t.\t" $9 }' ContScout/annotation_data/WFB1_full_canu_v0.1_br_v1.gff >> ContScout/annotation_data/output_with_genes.gff3
awk 'BEGIN {OFS="\t"} { print $0";"$NF }' ContScout/annotation_data/output_with_genes.gff3 | awk 'BEGIN {FS=OFS="\t"} {sub(/;[^;]+$/, "", $9)} 1'| sed "s/;ID=/;protein_id=/" output_with_genes_1.gff3
rm ContScout/annotation_data/output_with_genes.gff3 
rm ContScout/annotation_data/WFB1_full_canu_v0.1_br_v1.gtf

### Run ContScout against the Fomitiporia taxon ID from NCBI.
ContScout --userdir /DATA9/Projects/Basidios_genomes/Databases -d uniprotKB -i ContScout -q 251363 -a diamond -m 90G -t ContScout/tmp/
#### No contamination was found.

# In silico PCRs were made with multiple primers from bacteria, fungi, and plants. Only fungal ITS produced amplicons. They were blasted against the NCBI nucleotide collection matching *F. polymorpha*.
perl ./in_silico_PCR.pl -s WFB1_25.contigs.P1.fasta -a TCCGTAGGTGAACCTGCGG -b TCCTCCGCTTATTGATATGC > WFB1_25.contigs.P1__ITS_amplicons.txt 2> WFB1_25.contigs.P1__ITS_amplicons.fasta


# A gene copy number analysis was made to check genome structure. For this, OrthoFinder v.2.5.4 was used with the predicted proteins from *F. polymoprpha* and the diploid genome of *F. mediterranea*. (Check the "repeat and gene annotation section" to see the code used).

cp Annotation/WFB1_full_canu_v0.1_br_v1_PROT.fasta WFB1_vs_Fmed_prot/
cp Annotation/Fmed_full_canu_v0.1_br_v1_PROT.fasta WFB1_vs_Fmed_prot/

orthofinder -f WFB1_vs_Fmed_prot/ -o OrthoFinder/ -n comp_fom -t 10 -a 10 &

### Get the gene copy number. This was used to produce the plots of gene copy number variation.
cd WFB1_vs_Fmed_prot/OrthoFinder/Results_comp_fom/WorkingDirectory
zcat Blast0_0.txt.gz | cut -f1  | uniq -c | tr -s " " | tr " " "\t" | cut -f 2,3 | sort -n -k1,1 > Fmed.blast.all.counts.txt
zcat Blast1_1.txt.gz | cut -f1  | uniq -c | tr -s " " | tr " " "\t" | cut -f 2,3 | sort -n -k1,1 > WFB1.blast.all.counts.txt


#The results from the gene copy number variation suggested that the F. polymorpha genome was an incomplete diploid assembly or a possible aneuploid; therefore, manual curation was done.
```

### *F. polymorpha* genome assembly manual curation (primary and haplotigs)

```
# After discarding the possibility of contamination and finding evidence for a possible incomplete genome assembly, a manual curation was performed with BLAST+ 2.15.0 and MCScanX.

### Align proteins to themselves.
makeblastdb -in WFB1_full_canu_v0.1_br_v1_PROT.fasta -out WFB1_full_canu_v0.1_br_v1_PROT.fasta -dbtype prot 

blastall -p blastp -i WFB1_full_canu_v0.1_br_v1_PROT.fasta -d WFB1_full_canu_v0.1_br_v1_PROT.fasta -e 1e-10 -b 5 -v 5 -m 8 -o MCscanX/WFB1_full_canu_v0.1_br_v1_PROT.blast -a 8

### Prepare bed files from the gtf obtained at annotation.
cat WFB1_full_canu_v0.1_br_v1.gtf | grep -P "transcript\t" |  awk '{ print $9 "\t" $4 "\t" $5 "\t" $1}' | awk '{print $4 "\t" $1  "\t" $2 "\t" $3}' | sort -k1,1V -k3,3n | grep -v ".t2" > MCscanX/WFB1_full_canu_v0.1_br_v1_PROT.gff

### Run MCScanX with default parameters.
MCScanX MCscanX/WFB1_full_canu_v0.1_br_v1_PROT


### To get summary stats, the following script was used.

#!/bin/bash
################################################################################
# script                                                                         #
################################################################################
if [ "$1" = "" ] ;
  then
    echo Usage:  full/path/to/mcscanx/main/dir basename/of/mcscanx/results
  else

Dir=$1
basename=$2

cd $1/
cat $2.collinearity | grep "genes" | sed "s/ //g"| sed "s/:/\t/g" | sed "s/,/\t/g" | cut -f 2 -d "      " | sed "1s/^/$2\n/" > $2_syn_totals.txt

cat $2.collinearity | grep "Alignment" | sed "s/ /\t/g" | sed "s/&/\t/g" | cut -f 3,6,7,8,9 | less | sed "s/N=//g" | sed "1s/^/BlockNo\tCount_genes\tChr_a\tChr_b\tStrand\n/" > $2_syn_summary_table.txt

cat $2.gff | cut -f 1 | sort --version-sort  | uniq -c | tr -s ' ' | sed "s/ /\t/g" | awk '{print $2 "\t" $1 }' > $2_genes_per_scaff.txt

fi
################################################################################


### Then, contigs with more than 70% of the genes in colinear blocks with another contig were sent to a list of haplotigs and kept the rest to primary. This was done manually using the tables *syn_summary_table.txt and *genes_per_scaff.txt. Once these primary and haplotigs lists were generated, the fasta files were filtered accordingly using seqtk.

seqtk subseq WFB1_25.contigs.P1.fasta filter_mcscan_primary.txt > WFB1_25.contigs.P1_mcscanx_primary.fasta
seqtk subseq WFB1_25.contigs.P1.fasta filter_mcscan_haplotigs.txt > WFB1_25.contigs.P1_mcscanx_haplotigs.fasta
```

### Genome stats evaluation

```
# Genome completeness stats were calculated using BUSCO v.5.6.1 with the basidiomycota_odb10.

busco -i WFB1_25.contigs.P1_mcscanx_primary.fasta -l basidiomycota_odb10 -o BUSCO_WFB1_25_primary -m genome -c 20 & 
busco -i TX9_25_primary_PH.fasta -l basidiomycota_odb10 -o BUSCO_TX9_25_primary -m genome -c 20 & 
busco -i OC1_25_primary_PH.fasta -l basidiomycota_odb10 -o OC1_TX9_25_primary -m genome -c 20 & 
busco -i Fmed_v0.1.hap1.fasta -l basidiomycota_odb10 -o Fmed_v0.1.hap1 -m genome -c 20 & 

# A custom script was used to calculate overall stats from the genome assemblies. This script was called assembly_statistics.sh, and it was used as follows.

for genome in $(cat genomes.list.txt); do ./assembly_statistics.sh 90000000 ${genome}.fasta | tee ${genome}.stats ; done

# The content of the script is the following:

#!/bin/bash
script_dir=$(dirname $0)
genome_size=$1

function length_distribution {
# distribution of sequence lengths and assembled bases.
        echo Sequences\>100bp $(awk -F " " '$2>100' $1 | wc -l ) $(awk -F " " '$2>100' $1 |  awk '{ SUM += $2} END { print SUM}' )bp
        echo Sequences\>200bp $(awk -F " " '$2>200' $1 | wc -l ) $(awk -F " " '$2>200' $1 |  awk '{ SUM += $2} END { print SUM}' )bp
        echo Sequences\>500bp $(awk -F " " '$2>500' $1 | wc -l ) $(awk -F " " '$2>500' $1 |  awk '{ SUM += $2} END { print SUM}' )bp
        echo Sequences\>1Kbp $(awk -F " " '$2>1000' $1 | wc -l ) $(awk -F " " '$2>1000' $1 |  awk '{ SUM += $2} END { print SUM}' )bp
        echo Sequences\>2Kbp $(awk -F " " '$2>2000' $1 | wc -l ) $(awk -F " " '$2>2000' $1 |  awk '{ SUM += $2} END { print SUM}' )bp
        echo Sequences\>5Kbp $(awk -F " " '$2>5000' $1 | wc -l ) $(awk -F " " '$2>5000' $1 |  awk '{ SUM += $2} END { print SUM}' )bp
        echo Sequences\>10Kbp $(awk -F " " '$2>10000' $1 | wc -l ) $(awk -F " " '$2>10000' $1 |  awk '{ SUM += $2} END { print SUM}' )bp
        echo Sequences\>20Kbp $(awk -F " " '$2>20000' $1 | wc -l ) $(awk -F " " '$2>20000' $1 |  awk '{ SUM += $2} END { print SUM}' )bp
        echo Sequences\>50Kbp $(awk -F " " '$2>50000' $1 | wc -l ) $(awk -F " " '$2>50000' $1 |  awk '{ SUM += $2} END { print SUM}' )bp
        echo Sequences\>100Kbp $(awk -F " " '$2>100000' $1 | wc -l ) $(awk -F " " '$2>100000' $1 |  awk '{ SUM += $2} END { print SUM}' )bp
        echo Sequences\>200Kbp $(awk -F " " '$2>200000' $1 | wc -l ) $(awk -F " " '$2>200000' $1 |  awk '{ SUM += $2} END { print SUM}' )bp
        echo Sequences\>500Kbp $(awk -F " " '$2>500000' $1 | wc -l ) $(awk -F " " '$2>500000' $1 |  awk '{ SUM += $2} END { print SUM}' )bp
        echo Sequences\>1Mbp $(awk -F " " '$2>1000000' $1 | wc -l ) $(awk -F " " '$2>1000000' $1 |  awk '{ SUM += $2} END { print SUM}' )bp
        echo Sequences\>2Mbp $(awk -F " " '$2>2000000' $1 | wc -l ) $(awk -F " " '$2>2000000' $1 |  awk '{ SUM += $2} END { print SUM}' )bp
        echo Sequences\>5Mbp $(awk -F " " '$2>5000000' $1 | wc -l ) $(awk -F " " '$2>5000000' $1 |  awk '{ SUM += $2} END { print SUM}' )bp
        echo Sequences\>10Mbp $(awk -F " " '$2>10000000' $1 | wc -l ) $(awk -F " " '$2>10000000' $1 |  awk '{ SUM += $2} END { print SUM}' )bp
}

function gap_statistics {
# distribution of lengths
        gaps=${1}.gaps
        awk '$5=="N"' $1 | cut -f 1,6 > $gaps
        awk 'BEGIN {sum=0; num=0} { sum=sum+$2; num++  } END {print "Number_of_gaps\t"num",Number_of_Ns\t"sum",Average_gap_length\t"sum/num }' $gaps | tr "," "\n";
        echo -e "Median_gap_length\t$(cut -f 2 $gaps | Rscript -e 'd<-scan("stdin", quiet=TRUE); cat(median(d))')"
    length_distribution $gaps | sed 's: :\t:g'
}

function statistics {
        echo
        ${script_dir}/contigs_statistics.exe $1 | sed 's: :_:g;s:contigs:sequences:g'
        echo
        python ${script_dir}/nucleotide_content.py $1
        echo
        ${script_dir}/getLengthFromFasta.py $1 > ${1}.len
    echo
    echo -e "Median_seqence_length\t$(cut -f 2 ${1}.len | Rscript -e 'd<-scan("stdin", quiet=TRUE); cat(median(d))')"
        echo
    length_distribution ${1}.len | sed 's: :\t:g'
        echo
        ${script_dir}/contigs_ng.py $genome_size $1
}

export file=$2

echo "**************** $file statistics ****************"
echo

        # Global
        echo "******** Global statistics ********"
            statistics $file

    ${script_dir}/fastaToAGP.pl -i $file -o ./ -name ${file}.splitted >err.log 2>err.log

    echo
    echo "******** Gaps statistics ********"
                # Gaps in scaffolds
                gap_statistics ${file}.splitted.agp


        # Split in Contigs
    echo
        echo "******** Contig statistics ********"

        # Contigs
                statistics ${file}.splitted.contigs.fa
        echo
```

# Gene annotation

### Repeat modeling

```
# Model the repeats per species using RepeatModeler v.1.0.8 using the primary or haplotype 1.
for i in $(cat CLR_genome_id_list.txt); do RepeatModeler-open-1.0.8/BuildDatabase -name ${i}  ${i}_primary_*.fasta; done &
RepeatModeler-open-1.0.8/BuildDatabase -name Fmed_v0.1  Fmed_v0.1.hap1.fasta &
for i in $(cat genome_id_list.txt); do RepeatModeler-open-1.0.8/RepeatModeler -database ${i} -pa 24; done &

# Concat the predicted models per species with the repeat libraries from repbase library 20160829-2023.
for i in $(cat genome_id_list.txt); do cat RM_${i}/consensi.fa RepeatMasker/Libraries/RepeatMasker.lib > ${i}-repbase.repeat.noUnknown.20160829_2023.lib ; done

# Run RepeatMasker v.4.06 on primary and haplotigs (or in haplotypes 1 and 2 in F. mediterranea).

for i in $(cat CLR_genome_id_list.txt); do RepeatMasker/RepeatMasker -norna -lib ${i}-repbase.repeat.noUnknown.20160829_2023.lib -pa 24 -dir ${i}_R_masked/ ${i}_25_primary_*.fasta -gff ; done &

for i in $(cat CLR_genome_id_list.txt); do RepeatMasker/RepeatMasker -norna -lib ${i}-repbase.repeat.noUnknown.20160829_2023.lib -pa 24 -dir ${i}_R_masked/ ${i}_25_haplotigs_*.fasta -gff ; done &

for i in hap1 hap2; do RepeatMasker/RepeatMasker -norna -lib Fmed_v0.1-repbase.repeat.noUnknown.20160829_2023.lib -pa 24 -dir Fmed_v0.1_R_masked/ Fmed_v0.1.${i}.fasta -gff; done &

# Soft mask the repeats 
for i in $(cat CLR_genome_id_list.txt); do maskFastaFromBed -soft -fi ${i}_25_primary_*.fasta -fo ${i}_25_primary_*_softmasked.fasta -bed ${i}_R_masked/${i}_25_primary_*.fasta.out.gff ; done &

for i in $(cat CLR_genome_id_list.txt); do maskFastaFromBed -soft -fi ${i}_25_haplotigs_*.fasta -fo ${i}_25_haplotigs_*_softmasked.fasta -bed ${i}_R_masked/${i}_25_haplotigs_*.fasta.out.gff ; done &

for i in hap1 hap2; do maskFastaFromBed -soft -fi Fmed_v0.1.${i}.fasta -fo Fmed_v0.1.${i}_softmasked.fasta -bed Fmed_v0.1_R_masked/Fmed_v0.1.${i}.fasta.out.gff ; done &
```

### Gene prediction

```
# The genes were predicted using Braker v2.1.6 and the OrthoDB11 database of fungi downloaded from [https://bioinf.uni-greifswald.de/bioinf/partitioned_odb11/]

for i in $(cat CLR_genome_id_list.txt); do braker.pl --genome=Full/path/${i}_25_primary_*_softmasked.fasta --prot_seq=Full/path/Annotation/db_OrthoDB_v11/Fungi.fa --fungus --cores 20 --softmasking 1 --species=${i}_primary --overwrite --useexisting --GENEMARK_PATH=Full/path/programs/gmes_linux_64/ --PROTHINT_PATH=Full/path/programs/ProtHint/bin --BAMTOOLS_PATH=Full/path/programs/bamtools/bin; done &

for i in $(cat CLR_genome_id_list.txt); do braker.pl --genome=Full/path/${i}_25_haplotigs_*_softmasked.fasta --prot_seq=Full/path/Annotation/db_OrthoDB_v11/Fungi.fa --fungus --cores 20 --softmasking 1 --species=${i}_haplotigs --overwrite --useexisting --GENEMARK_PATH=Full/path/programs/gmes_linux_64/ --PROTHINT_PATH=Full/path/programs/ProtHint/bin --BAMTOOLS_PATH=Full/path/programs/bamtools/bin; done &

for i in hap1 hap2; do braker.pl --genome=Full/path/Fmed_v0.1.${i}_softmasked.fasta --prot_seq=Full/path/Annotation/db_OrthoDB_v11/Fungi.fa --fungus --cores 20 --softmasking 1 --species=Fmed_v0.1.${i} --overwrite --useexisting --GENEMARK_PATH=Full/path/programs/gmes_linux_64/ --PROTHINT_PATH=Full/path/programs/ProtHint/bin --BAMTOOLS_PATH=Full/path/programs/bamtools/bin; done &


# To clean the annotation, different premade and custom scripts were used. For space purposes, the steps will be shown for only one file, which was used for the primary and haplotigs (or hap1 and hap2) of all the species.  
  
### To get mRNA from the GFT file, getAnnoFasta.pl was used, a premade script within Augustus.
getAnnoFasta.pl --seqfile=Full/path/TX9_25_primary_PH_softmasked.fasta Braker_output/braker.gtf &

### Translate mRNA file into amino acids.
mkdir parsed
transeq -sequence braker.mrna -outseq parsed/braker_to_parse.aa

# Gathering IDs to remove.
cd parsed
grep ">" braker_to_parse.aa | grep -v t1 > non_t1_seqIDs
fasgrep -vs '\*$' braker_to_parse.aa | grep ">" > non_stop_end_seqIDs
fasgrep -sv ^M braker_to_parse.aa | grep ">" > non_start_codon_seqIDs
fasgrep -s '\*' braker_to_parse.aa | fasgrep -vs '\*$' | grep ">" > internal_stops_seqIDs
cat *seqIDs | tr -d '>' | sort | uniq | sed 's/.t1//' > to_remove_IDs

# Removing lines in gtf.
grep -v -w --file=to_remove_IDs ../braker.gtf > braker.parsed.gtf
grep "AUGUSTUS" braker.parsed.gtf| sed 's/file_1_file_1_/TX9\./g' > braker.parsed_renamed.gtf

# Creating final gtf and amino acids file.
getAnnoFasta.pl --seqfile=Full/path/TX9_25_primary_PH_softmasked.fasta braker.parsed_renamed.gtf

cd ../
mkdir final_files

# Copying and changing to final names.
cp parsed/braker.parsed_renamed.codingseq final_files/TX9_canu_v0.1_br_v1_CDS_primary.fasta
cp parsed/braker.parsed_renamed.gtf final_files/TX9_canu_v0.1_br_v1_primary.gtf
cd final_files/
transeq -sequence TX9_canu_v0.1_br_v1_CDS_primary.fasta > TX9_canu_v0.1_br_v1_PROT_primary.fasta
```

### Gene and repeat density

```
# To get the gene and repeat density, the bedtools package was used. The primary and haplotigs (or hap1 and hap2) were concatenated and processed together. For space purposes, the steps will be shown for only one species, but this was used for all the species.

### Convert the repeats gff to bed using convert2bed.
convert2bed --input=gff < TX9_25_all_PH_out_repeats.gff | sed s/^/${i}\./1 | awk '{print $1,$2,$3,$11,$7}' OFS="\t" | sort -V | sed 's/\"//g' | sed s/Motif://g  > TX9_25.all_PH_repeats.bed

### Convert the genes gff to bed using gff2bed.
gff2bed < TX9_canu_v0.1_br_v1_all.gtf | grep "mRNA" | cut -f 1-4 > TX9_25.all_PH.gene.bed

### Get chr size from genome.fasta.
samtools faidx TX9_25.all_PH.fasta && awk '{print $1"\t0\t"$2}' TX9_25.all_PH.fasta.fai > TX9_25.all_PH_chr.bed && cut -f1,2 TX9_25.all_PH.fasta.fai > TX9_25.all_PH_chr_size.tab

### Create 10kbp Windows using bedtools.
bedtools makewindows -g TX9_25.all_PH_chr_size.tab -w 10000 > TX9_25.all_PH_chr.windows10kb

### Density calculation using bedtools.
bedtools coverage -b TX9_25.all_PH.gene.bed -a TX9_25.all_PH_chr.windows10kb | sed "s/^/${i}\tgene\t/1" > TX9_25.all_PH_gene_density
bedtools coverage -b TX9_25.all_PH_repeats.bed -a TX9_25.all_PH_chr.windows10kb | sed "s/^/${i}\trepeat\t/1" > TX9_25.all_PH_repeat_density
```

# Functional Annotation

```
# For space purposes, the steps will be shown for only one species, which was used for all the species.

### Pfam domain annotations was made with PfamScan and the Pfam-A database.
PfamScan/pfam_scan.pl -fasta Full/path/TX9_canu_v0.1_br_v1_PROT.fasta -dir /PfamScan/lib -as -cpu 8 -e_seq 0.001 -e_dom 0.001 -outfile pfam/TX9.Pfam.v3.1.out

cat  pfam/TX9.Pfam.v3.1.out | tail -n +29 | tr -s ' ' | sed 's/ /\t/g' | awk '{FS="\t" ; OFS="\t"} {print $1,$6,$7,$8}' | tail -n +2 | awk 'BEGIN {getline ; ID=$1 ; line=$2} {if ($1==ID) { line=line"+"$2} else { print ID,line ; ID=$1 ; line=$2} } END { print ID, line } ' | tr " " "\t" > pfam/TX9.1 ; done

cat pfam/TX9.Pfam.v3.1.out | tail -n +29 | tr -s ' ' | sed 's/ /\t/g' | awk '{FS="\t" ; OFS="\t"} {print $1,$6,$7,$8}' | tail -n +2 | awk 'BEGIN {getline ; ID=$1 ; line=$3} {if ($1==ID) { line=line"+"$3} else { print ID,line ; ID=$1 ; line=$3} } END { print ID, line } ' | cut -f2 -d " " > pfam/TX9.2 ; done

paste TX9.1 TX9.2 > TX9.Pfam.v3.1.parsed_final.txt


### The signal peptides were assigned using SignalP 5.0. 
signalp-5.0/bin/signalp -batch 40000 -fasta Full/path/TX9_canu_v0.1_br_v1_PROT.fasta -org euk -format short -prefix SignalP/TX9_SignalP

cat SignalP/TX9_SignalP_summary.signalp5 | tail -n +3 | grep -v "OTHER" > SignalP/TX9_signalp5_parsed.txt


### CAZymes were annotated with the dbCAN3 at [https://bcb.unl.edu/dbCAN2/blast.php](https://bcb.unl.edu/dbCAN2/blast.php) selecting the options “HMMER: dbCAN (E-Value < 1e-15, coverage > 0.35)”, “DIAMOND: CAZy (E-Value < 1e-102)” and “HMMER: dbCAN-sub (E-Value < 1e-15, coverage > 0.35)”. The annotation was kept only when the genes were annotated with at least two algorithms. 
#### Genes with Signal peptides and CAZymes were annotated as secreted CAZymes.


### Cytochrome P450s were annotated using Phmmer,  diamond blast against the CYPED 6 database. The Pfam annotation was used too. Genes with annotation with at least two methods were kept as P540.
hmmer-3.2.1/src/phmmer -E 0.001 --incE 0.001 -o P450/TX9_tb1.txt --tblout P450/TX9_tb2.txt --noali --seed 42 --cpu 20 Full/path/TX9_canu_v0.1_br_v1_PROT.fasta db/cyped.p540_db.fa

diamond blastp -d db/cyped.p540_db.dmnd -q Full/path/TX9_canu_v0.1_br_v1_PROT.fasta -o P450/TX9_diamond_blastp_results.txt --id 60 --outfmt 6 qseqid qlen sseqid slen pident length mismatch gapopen evalue bitscore --evalue 0.0001 --threads 10


### Fungal peroxidases were annotated using hmmsearch HMMER v.3.1b2 and the hmm models of fPoxDB.
for i in {1..25}; do hmmsearch -E 1e-5 --tblout Peroxidases/${i}.TX9.fPoxDB.outp ${i}.hmm.ASCII Full/path/TX9_canu_v0.1_br_v1_PROT.fasta ; done


### Secondary metabolite clusters were annotated using antiSMASH v.6.0 at [https://fungismash.secondarymetabolites.org] with default parameters. The genes in each cluster were given the annotation of the cluster to which they belonged.

### The Transporters were annotated using Diamond blastp with the option against the TCDB database.
diamond blastp -d db/tcdb_march_2023.dmd -q Full/path/TX9_canu_v0.1_br_v1_PROT.fasta -evalue 1e-5 --threads 10 -o Transporters/TX9_TCDB.blast.out
```

# Phylogenetic analysis

### Maximum likelihood tree

```
# Aditional genomes for phylogenetic reference and calibration were retrieved from different repositories. The species, isolate, and reference of these additional genomes are reported in Supplementary Table 1.  
  
# The predicted proteins of all the genomes were used as input for OrthoFinder v.2.5.4. All the protein files were copied to a directory called Phylogeny, and OrthoFinder was run with default parameters.
orthofinder -f Phylogeny/ -o OrthoFinder_all/ -n comp_all -t 20 -a 20 &

# Single Copy Orthologues (SCO) were used for Phylogeny constructions. First, the SCOs were aligned using MUSCLE v.5.1.
cd Phylogeny/OrthoFinder_all/Results_comp_all/Single_Copy_Orthologue_Sequences/
mkdir alignments/
for i in $(ls -1 OG*); do muscle -in ${i} -maxiters 16 -out alignments/${i}.aln ; done &

# Convert the fasta alignments to tab, split alignment files per species, and paste the sequences in a single line. Then, concatenate all the species into a single fasta file.
cd alignments/
for i in $(ls *fa.aln); do awk '/^>/{if(NR>1)printf("\n%s\t", substr($0,2));else printf("%s\t", substr($0,2));next}{printf "%s",$0}END{print ""}' ${i} > ${i}.tab ; done

for i in $(ls -1 *.aln.tab); do for z in $(cat species.list.ids); do grep "${z}" ${i} | cut -f 2 | tr --delete '\n' >> ${z}_concat.tab ; done ; done

for i in $(cat species.list.ids); do awk '{ print $1}' ${i}_concat.tab ; done > all_concat.txt

paste species.list.ids all_concat.txt | sed "s/^/>/" | tr "\t" "\n" > all_concat.fasta


# The fasta alignment files were cleaned to get phylogenetically informative regions using Gblocks_0.91b in interactive mode.
Gblocks_0.91b/Gblocks # then, import the next file
Phylogeny/OrthoFinder_all/Results_comp_all/Single_Copy_Orthologue_Sequences/alignments/all_concat.fasta # then, use default parameters get the blocks.

# The Gblocks cleaned alignments were used for Phylogenetic model prediction using ModelTest-NG v.0.1.7.
modeltest-ng -d aa -i Phylogeny/OrthoFinder_all/Results_comp_all/Single_Copy_Orthologue_Sequences/alignments/Phylogeny/all_concat_gb0.fasta -f f -h uigf -o modeltest.all -p 20 -r 1234 -m JTT,LG,WAG

# The optimized model was used to calculate the Maximum Likelihood Phylogenetic tree using RAxML-NG v.0.9.0.
raxml-ng --all --msa all_concat_gb0.fasta --seed 12345 --tree pars{10} --bs-trees 100 --threads 20 --model LG+I+G4

# The resulting tree was visualized and prepared using Figtree.
```

### Clock-calibrated tree

```
# The clock-calibrated tree was constructed using BEAST v2.7.6 and included packages. To prepare the xml file required for BEAST, the interactive program BEAUti was used. To do this, the following steps were followed.  
  
##1. The cleaned alignment of single-copy orthologs was imported into BEAUti.  
##2. Monophyletic partitions were created for Ascomycetes and the Polyporales group.  
##3. Calibration points for these partitions were set based on the literature. Ascomycetes crown to 539 Mya (Prieto and Wedin 2013), and the Polyporales group set to 142 Mya (Ji et al. 2022), with a normal distribution.  
##4. The LG substitution model with four gamma categories, a strict clock, and the Birth-Death model was selected.  
##5. Five different Markov chain Monte Carlo chains of 1,000,000 generations were set.  
  
# The file was saved to run with BEAST.
for i in a b c d e ; do beast -overwrite -threads 10  Basidios_gb0_BB_BD_${i}.xml ; done

# The resulting log and tree files were combined using LogCombiner v.2.7.6, and the maximum credibility tree used TreeAnnotator v2.7.6 with a burn-in of 10,000 generations.  
  
# Figtree was used to visualize and prepare the phylogenetic tree.
```

# Gene family expansion and contraction analysis

### CAFE analysis

```
# The predicted proteins in all the genomes were concatenated in a single file and blasted to themselves using blastp within Diamond v2.1.8.162.

cat prot/*.fasta > CAFE/all_set.fa

diamond makedb --in CAFE/all_set.fa  -d CAFE/all_set.db &
diamond blastp -q CAFE/all_set.fa -d CAFE/all_set.db --evalue 1e-6 -o CAFE/diamond/all_vs_all.tsv --threads 20 --very-sensitive --outfmt 6 qseqid sseqid evalue

# The proteins were grouped in families using Markov clustering with MCL v.14-137.
mcl-14-137/bin/mcxload -abc CAFE/diamond/all_vs_all.tsv --stream-mirror --stream-neg-log10 -stream-tf 'ceil(200)' -o CAFE/mcl/all_vs_all.mci -write-tab CAFE/mcl/all_vs_all.tab

mcl-14-137/bin/mcl CAFE/mcl/all_vs_all.mci -I 3 -o CAFE/mcl/Out_all_vs_all.mci.I30 

mcl-14-137/bin/mcxdump -icl CAFE/mcl/Out_all_vs_all.mci.I30 -tabr CAFE/mcl/all_vs_all.tab -o CAFE/mcl/dump.Out_all_vs_all.mci.I30

# Prepare CAFE input with a script found in [https://github.com/hahnlab/cafe_tutorial/tree/main/python_scripts]
python CAFE_python_scripts/cafetutorial_mcl2rawcafe.py -i CAFE/mcl/dump.Out_all_vs_all.mci.I30 -o CAFE/mcl/all_vs_all.mci.I30.CAFE.input.txt -sp "Bot_cin Bot_dot Dae_que Fom_med Fom_pol Fom_sch Fus_oxy Glo_tra Ino_vit Neo_par Pha_chl Ple_ost Pos_pla Sac_cer Ser_lac Ste_hir Tra_ver Tro_tex"

## Convert the clock-calibrated tree to newick format with node age using https://itol.embl.de.

# CAFE was run with the clock calibrated tree and CAFE/mcl/all_vs_all.mci.I30.CAFE.input.txt
cafe5 -i CAFE/mcl/all_vs_all.mci.I30.CAFE.input.txt -t Basidios_BB_BD_newick.txt -c 30 -o CAFE/cafe/all_vs_all.mci.I30.CAFE -P 0.0100 &

# Filter the input file to keep only the families undergoing significant change.
grep -v "n" CAFE/cafe/all_vs_all.mci.I30.CAFE/Base_family_results.txt | cut -f 1 > CAFE/cafe/all_vs_all.mci.I30.CAFE/sig.fams

{ head -n 1 CAFE/mcl/all_vs_all.mci.I30.CAFE.input.txt; for i in $(cat CAFE/cafe/all_vs_all.mci.I30.CAFE/sig.fams); do grep -P "null\)\t${i}\t" CAFE/mcl/all_vs_all.mci.I30.CAFE.input.txt; done; } > CAFE/mcl/Sig_all_vs_all.mci.I30.CAFE.input.txt

# With the filtered input file, rerun CAFE with a fixed lambda (calculated in the previous run).
cafe5 -i CAFE/mcl/Sig_all_vs_all.mci.I30.CAFE.input.txt -t Basidios_BB_BD_newick.txt -l 0.0011169463915058 -c 30 -o CAFE/cafe/all_vs_all.mci.I30.CAFE/hard_filetred/ -P 0.0100 &
```

### CAFE results parsing

```
cd CAFE/

# Add family number id.
nl -w1 -s$'\t' mcl/dump.Out_all_vs_all.mci.I30 > mcl/fam_gene_compact.txt

# Parse families file to have one gene per line (from compact to large).
awk '{id=$1; $1=""; for(i=2;i<=NF;i++) print id,$i}' mcl/fam_gene_compact.txt > mcl/fam_gene_large.txt

# Parse the Base_change to keep only  tips nodes.
cut -f 1-19 cafe/all_vs_all.mci.I30.CAFE/hard_filetred/Base_change.tab | sed 's/<[^>]*>//g' > cafe/all_vs_all.mci.I30.CAFE/hard_filetred/Base_change.txt

# Splits files by columns, keeping the column id as filename.
cd cafe/all_vs_all.mci.I30.CAFE/hard_filetred/

awk -F'\t' 'NR==1{split($0, cols, "\t")} {for(i=1; i<=NF; i++) print $i > cols[i]".txt"}' Base_change.txt

# Create a file with node IDs (isolates).
head -n1 Base_change.txt | cut -f 2-19 | tr "\t" "\n" > species


# Paste families IDs with change if the value is negative = Contracted,  if positive = Expanded. Remove 0s.
cd ../../../

for i in  $(cat cafe/all_vs_all.mci.I30.CAFE/hard_filetred/species ); do paste cafe/all_vs_all.mci.I30.CAFE/hard_filetred/FamilyID.txt cafe/all_vs_all.mci.I30.CAFE/hard_filetred/${i}.txt | grep -v -P '\t0$' | awk 'BEGIN{FS=OFS="\t"} NR>1 { $2 = ($2 < 0) ? "Contracted" : "Expanded"; print }' | sort -t $'\t' -k1,1n > cafe/all_vs_all.mci.I30.CAFE/hard_filetred/${i}.fam_change.txt; done


# Split the genes in each family by isolate.
for i in $(cat cafe/all_vs_all.mci.I30.CAFE/hard_filetred/species); do grep "${i}" mcl/fam_gene_large.txt | tr " " "\t" | sort -t $'\t' -k1,1n > mcl/${i}_fam_genes.txt; done


# Join Family ID + Gene + Direction + Isolate ID.
for i in $(cat cafe/all_vs_all.mci.I30.CAFE/hard_filetred/species); do awk -v var="$i"  'BEGIN { FS = OFS = "\t" } NR == FNR { data[$1] = $2; next } $1 in data { print $1, $2, data[$1], var } !($1 in data) { print $1, "not_found", var }' cafe/all_vs_all.mci.I30.CAFE/hard_filetred/${i}.fam_change.txt mcl/${i}_fam_genes.txt | grep -v "not_found" > cafe/all_vs_all.mci.I30.CAFE/hard_filetred/${i}_fam_genes_direction.txt ; done


# The tree with the number of families under expansion and contraction was created using CafePlotter [https://github.com/moshi4/CafePlotter].
cafeplotter -i CAFE/cafe/all_vs_all.mci.I30.CAFE/hard_filetred/ -o ./basidios_plot --ignore_branch_length --format 'pdf' --innode_label_size 12


# A Fisher exact test was used to obtain the functions that were significantly enriched in the expanded and contracted families of the species of interest. These functions were plotted in wordclouds using [https://www.wordclouds.com].
```
